# Cryptotanshinone inhibits PFK-mediated aerobic glycolysis by activating AMPK pathway leading to blockade of cutaneous melanoma

**DOI:** 10.1186/s13020-024-00913-1

**Published:** 2024-03-07

**Authors:** Qiong Chen, Yang Liu, Yunxuan Zhu, Ziyan Zhu, Jueyao Zou, Yanhong Pan, Yin Lu, Wenxing Chen

**Affiliations:** 1 Jiangsu Key Laboratory for Pharmacology and Safety Evaluation of Chinese Materia Medica, School of Pharmacy, Nanjing University of Chinese Medicine, Nanjing, 210023 China; 2 Jiangsu Collaborative Innovation Center of Traditional Chinese Medicine (TCM) Prevention and Treatment of Tumor, Nanjing, China; 3 Department of Pharmacy, The Second Affiliated Hospital of Nanjing Medical University, Nanjing, China; 4 Jiangsu Joint International Research Laboratory of Chinese Medicine and Regenerative Medicine, Nanjing, China

**Keywords:** Cryptotanshinone, Aerobic glycolysis, Melanoma, PFK, AMPK, HIF-1α

## Abstract

**Background:**

Cutaneous melanoma is a kind of skin malignancy with low morbidity but high mortality. Cryptotanshinone (CPT), an important component of *salvia miltiorrhiza* has potent anti-tumor activity and also indicates therapeutic effect on dermatosis. So we thought that CPT maybe a potential agent for therapy of cutaneous melanoma.

**Methods:**

B16F10 and A375 melanoma cells were used for in vitro assay. Tumor graft models were made in C57BL/6N and BALB/c nude mice for in vivo assay. Seahorse XF Glycolysis Stress Test Kit was used to detect extracellular acidification rate and oxygen consumption rate. Si-RNAs were used for knocking down adenosine monophosphate-activated protein kinase (AMPK) expression in melanoma cells.

**Results:**

CPT could inhibit the proliferation of melanoma cells. Meanwhile, CPT changed the glucose metabolism and inhibited phosphofructokinase (PFK)-mediated glycolysis in melanoma cells to a certain extent. Importantly, CPT activated AMPK and inhibited the expression of hypoxia inducible factor 1α (HIF-1α). Both AMPK inhibitor and silencing AMPK could partially reverse CPT’s effect on cell proliferation, cell apoptosis and glycolysis. Finally, in vivo experimental data demonstrated that CPT blocked the growth of melanoma, in which was dependent on the glycolysis-mediated cell proliferation.

**Conclusions:**

CPT activated AMPK and then inhibited PFK-mediated aerobic glycolysis leading to inhibition of growth of cutaneous melanoma. CPT should be a promising anti-melanoma agent for clinical melanoma therapy.

**Supplementary Information:**

The online version contains supplementary material available at 10.1186/s13020-024-00913-1.

## Introduction

Cutaneous melanoma is the most serious skin cancer worldwide, and accounts for approximately twenty percent of skin cancers. About 1.5 million new cases with skin cancer were estimated in 2020. Among them, 325,000 patients were diagnosed with malignant melanoma [1]. Conventional surgical excision, chemotherapy and radiation have been used against malignant melanoma. However, the development of resistance and adverse toxicity limits their applications in clinic [2]. There has also been surgical resection combined chemotherapy in clinical practice, but the results are far from satisfaction. Therefore, it is very necessary to develop new anti-melanoma drugs with lower toxicity and higher efficacy [3].

*Salvia miltiorrhiza*, a famous Chinese medicine, is always used to treat cardiovascular diseases [4]. Afterwards, its anti-tumor activity has been found based on plenty of research [4]. Meanwhile, its effect on skin diseases has also been concerned. A clinical study indicated that a formula containing Salvia miltiorrhiza can decrease the risk of lupus nephritis in patients with systemic lupus erythematosus [5]. A lipophilic component from Salvia miltiorrhiza, tanshinone IIA can inhibit growth of keratinocytes through inducing cell cycle arrest and apoptosis to cure the psoriasis [6]. Another lipophilic compound CPT also indicates potential activity on skin diseases. CPT can ameliorate the pathogenesis of systemic lupus erythematosus by blocking T cell proliferation [7], reduce psoriatic epidermal hyperplasia via inhibiting the activation of STAT3 [8]. More importantly, CPT has been considered a potent anti-tumor natural compound with low toxicity [9, 10]. Therefore, we thought that CPT may have inhibitory effect on cutaneous melanoma.

Unlike normal cells, most tumor cells including melanoma cells show energy metabolism disorders that ATP production is from tumor cell glycolysis but not tricarboxylic acid cycle (TCA). The amount of ATP generated from glycolycis is significantly less than TCA, but the rate of ATP production is just contrary. The preferred energy metabolism way of tumor cells is named as aerobic glycolycis or Warburg effect [11]. Tumor cells undergo aerobic glycolysis, leading to a significant synthesis of ATP, enhancing the anti-oxidant and proliferative abilities of tumor cells [11]. Certainly, it was affected and regulated by many factors among which hypoxia inducible factor 1α (HIF-1α) is one of the most important factors in the hypoxic tumor microenvironment [12]. It activates the transcription of genes encoding proteins in the oxygen independent energy production pathway, directly promoting glycolysis in tumor cells. Meanwhile, glycolysis can promote the expression of HIF-1α by a feedback way. HIF-1α further promotes the proliferation of tumor cells and avoids cell apoptosis processes through its downstream signaling pathways, while HIF-1α in turn can directly promote the glycolysis [13]. Additionally, adenosine monophosphate-activated protein kinase (AMPK) always maintains intracellular energy homeostasis through increasing catabolism and reducing anabolism when low energy condition occurs [14]. Inhibiting AMPK activity increases the glycolytic metabolism of cancer cells, while silencing HIF-1α can reverse this metabolic transformation and reduce the proliferative advantage of cancer cells induced by AMPK inactivation [15].

In previous study, we have demonstrated that CPT inhibited mTORC1 leading to cell death via activating AMPK in Rh30 cells [16]. And the melanoma cells belong to the typical glycolytic cells, analyzing metabolism of melanoma cells can offer innovative insights in new anti-tumor targets for melanoma therapy [17]. Therefore, we hypothesized that CPT can inhibit cutaneous melanoma through activating AMPK and suppressing aerobic glycolysis, thereby affecting the proliferation and apoptosis of melanoma cells.

## Materials and methods

### Cell culture experiments

Established cell lines (A375, ATCC CCL-1619; B16F10, ATCC CRL-6475) were maintained in DMEM (Gibco) supplemented with 10% fetal bovine serum (FBS)(Cellmax). Cells are cultured in a constant temperature incubator with 5% CO_2_ and 37℃ conditions.

### Cell counting kit-8 assay

After the cells fully adhere to the wall, the supernatant was removed using a suction pump and different concentrations of CPT were added. After 24 h of cultivation, added the prepared alkaline medium containing 0.5 mg/mL MTT (Bioroxx, 334Gr001). After further cultivation at 37 °C for 4 h, 150 μL dimethyl sulfoxide was added to each well. Shaked the plate on a vibration table for 10 min. Then placed it in a microplate reader (BioTek) to measure the absorbance at 490 nm.

### Cell apoptosis and proliferation detection

BeyoClick™ EdU-555 Cell Proliferation Detection Kit (Beyotime Biotechnology, C0075S) was used to detect cell proliferation. Cells were pretreated with CPT. Followed the instructions for operation to observe and counted the EDU-positive cell rate under a fluorescence microscope (olympus, Stemi 2000C). The Beyotime Biotechnology (C1062M) assay kit was used for detecting cell apoptosis using membrane associated protein V-FITC. After cell administration, followed the instructions and used flow cytometry to detect the samples (C6, BD).

### Cell cycle experiment

The Cell Cycle and Apoptosis Detection Kit (C1052) were used to detect cell cycle. Followed the instructions for operation. Then added the newly prepared propidium iodide staining solution, leaved it in the dark at 37 °C for 30 min, and analyzed the DNA content using flow cytometry.

### Colony-formation assay

Transfected cells were collected and seeded into a 6-well plate with 200 cells/well. After conventional culture for 2 weeks, the culture was terminated when visible clones appeared in the 6-well plate. After PBS washing for two times, the cells were fixed by 4% methanol for 20 min, and then stained by 0.1% crystal violet for 15 min. The images were observed under an inverted phase contrast microscope (Stemi 2000C, Olympus) and the colonies were counted.

### Detection of metabolites

Cells underwent pre-treatment with drugs. Collected cells and added 500 µL of 75% methanol MTBE (9:1, v/v) containing 1,2-13C2 myristic acid (5 µg/mL) stored at − 20 °C per well. Collected cells into a 1.5 mL centrifuge tube using cell scraping and repeated freeze–thaw three times. Afterwards, centrifuged the sample and transferred 500 µL of the supernatant into a new 1.5 mL centrifuge tube. Used a vacuum concentrator to evaporate the sample at 45 °C. After the sample was evaporated, it was subjected to derivatization treatment. Joined 30 µL prepared methoxypyridine (w/v, 10 mg/mL), vortex for 5 min, and shaked for 1.5 h at 30 °C. Then added another 30 µL BSTFA (1% TMCS), vortex for 5 min, shaked for 0.5 h at 37 °C. After derivatization, the sample was centrifuged at 18,000 rpm at 4 °C for 10 min for subsequent loading. Sample analysis was performed using GC–MS (Trace 1310/TSQ 8000), and sample separation was performed at TG-5MS (30 m × 0.25 mm, 0.25 µm) on a capillary column.

### ECAR and OCR detection

Extracellular acidification rate (ECAR) and oxygen consumption rate (OCR) were examined using Seahorse XF Glycolysis Stress Test Kit and Seahorse XF Cell Mito Stress Test Kit, respectively. Briefly, 1 × 10^4^ cells per well were seeded into a Seahorse XF 96-cell culture microplate. On the day before boarding, added 200 µL of hydration solution to the lower layer of the XF96 Extracellar Flux Assay Kits. Placed it in a 37 °C CO_2_ free incubator for hydration overnight. Prepared the required drugs and hippocampal XF base medium, adjusted the pH value to 7.4 ± 0.1, and placed them in a 37 °C water bath for 1 h during use. On the second day, the cells were washed twice with water soaked hippocampal XF base medium. Finally, 175 µL of hippocampal XF base medium was added to each well and incubated at 37 °C in a CO_2_ free incubator for 1 h. Diluted the drug and added it to the upper layer of XF96 Extracellar Flux Assay Kits, with 25 µL added to each well before use. 30 min later, took out the lower layer of XF96 Extracellular Flux Assay Kits and replaced it with a cell plate that has been incubated for 1 h in a CO_2_ free incubator at 37 °C, and detected it on the Seahorse instrument.

### Pruvate, lactate, glucose uptake and ATP detection

Collected cells into a centrifuge tube and placed them in a tissue grinder for mechanical grinding. After grinding, centrifuged at 2500 rpm for 10 min, took the supernatant and operated according to the instructions of the pyruvate, lactate and glucose detection reagent kit. Then detected the OD value in a microplate reader (BioTek). Collected cells and used ATP to detect the lysate to lyse cells on ice for 30 min. Afterwards, centrifuged at 12,000 rpm for 5 min to remove the supernatant. Followed the instructions of the ATP detection kit and detected the chemiluminescence value in a microplate reader (BioTek).

### RNA extraction, PCR and real-time quantitative PCR

Total RNA was isolated from cells using Trizol and cDNA was synthesized using the HiScript® III-RT SuperMix kit (Vazyme, R323-01). The cDNA samples were subjected to a real-time quantitative polymerase chain reaction (RT-qPCR) using the ChamQ Universal SYBR qPCR Master Mix (Vazyme, Q711-02) performed on an ABI 7500 Sequence Detection System (Thermo Fisher). The primer sequences were listed as follow.

**Table:** 

ACTB	Forward	GGCTGTATTCCCCTCCATCG
	Reverse	CCAGTTGGTAACAATGCCATGT
HK2	Forward	ATGATCGCCTGCTTATTCACG
	Reverse	CGCCTAGAAATCTCCAGAAGGG
PFKFB3	Forward	TCTGGATGCCGTACAGCAATG
	Reverse	GTGTCGGACAGTTAGTCATGC
PKM2	Forward	CGCCTGGACATTGACTCTG
	Reverse	GAAATTCAGCCGAGCCACATT
LDHA	Forward	CAAAGACTACTGTGTAACTGCGA
	Reverse	TGGACTGTACTTGACAATGTTGG
MCT4	Forward	CACGGGTTTCTCCTACGCC
	Reverse	GCTGTAGCCAATCCCAAACTC

### Cell extracts and western blot analysis

Proteins were collected from cells and mice tissues by using RIPA lysis buffer. After separation by SDS-PAGE, proteins were transferred to PVDF membranes and detected with specific antibodies. Antibodies specific for anti-HK2 (ABclonal, A0994), anti-PFKFB3 (ABclonal, A3934), anti-AMPK (ABclonal, A1229), anti-p-AMPK (ABclonal, AP1002), anti-HIF-1α (ABclonal, A22041), anti-PKM2 (Abcam, ab89364), anti-LDHA (Affinity, DF6280), anti-MCT4 (Affinity, AF5253), and anti-β-actin (Affinity, AF7018) were used. The relative protein expression was determined by an imaging system (ChemiDoc TM XRS+, Bio-Rad).

### Immunofluorescence

Tumor spheroids were placed in 4% paraformaldehyde and then processed with Triton X-100 for 20 min and incubated respectively with 5% bovine serum albumin and anti-PFKFB3 (ABclonal, A3934) and anti-p-AMPK (ABclonal, AP1002) antibody overnight. Next day, the spheroids were rinsed thrice and cultivated in secondary antibodies (Invitrogen, Alexa flour-594, Invitrogen) for 2 h at indoor temperature. The cell nucleus was used by using Hoechst33342 (Beyotime, 1810898). Images were acquired using a confocal microscope (Zeiss, Vert.A1).

### Immunohistochemical and TUNEL

Mouse xenograft primary tumors and lung tissues were paraffin-embedded and sectioned by routine techniques. Deparaffinization and rehydration of tissue sections were first achieved. Heat-induced antigen retrieval was done using Decloaker solution for 15–20 min (Biocare Medical, RD913L). Tissue sections were blocked with TBS/10% NGS, then incubated with primary antibody overnight, followed by Dako envision plus kit and DAB staining. All samples were counterstained with hematoxylin. Samples were photographed using a multifunctional pathological imaging system (PerkinElmer, Mantra 1.0.1).

The pre-treatment was the same as before. Used TUNEL reagent kit for component staining. Subsequently, according to the manufacturer’s instructions, TUNEL was performed using the in situ cell death detection kit (Roche, Penzberg, Germany). Afterwards, took photos using a fluorescence microscope (Zeiss, Vert.A1).

### Si-RNA interference

Si-RNA against human AMPK (EKBIO) were introduced into cells by lipid mediated transfection using si-RNA transfection medium, reagent and duplex (Santa Cruz biotechnology) following manufacturer recommendations. Briefly the day before transfection cells were platted at 2.5 × 10^5^ cells per well in 2 mL antibiotic-free normal growth medium supplemented with FBS. Cells were incubated until they reach 60–80% confluence. The duplex solution containing the si-RNA was then added to the cells. After 5 to 7 h, antibiotic were added in each well and the cells were incubated for 24 h more. The media was then replaced by normal growth media and cells were used for experiments and assayed by western blot to analyze the expression of AMPK.

### Tumor graft models in mice

The allograft tumor model was made using B16F10 cells in male C57BL/6N mice (4–5 weeks old, obtained from Shanghai Slac Laboratory Animal CO.LTD). Adjust the concentration of B16F10 cells to 1.5 × 10^6^/mL. Inoculate 0.1 mL of cell suspension under the right back skin of male mice. After about two weeks, the protruding tumor tissue on the back of the mice can be clearly observed, and the mice will be randomly divided into groups. On the second day after grouping, medication was administered by gavage at a volume of 0.1 mL/10 g, while the model group was given an equal amount of olive oil. The length and width of the solid tumor were measured every two day using the caliper. At 14-day post-treatment, all mice were sacrificed, the tumor tissues were collected for corresponding assays. The tumor volume was calculated according the formula length × width^2^ × 0.52. The xenograft tumor model was repeated using A375 cells in male BALB/c nude mice (4–5 weeks old, obtained from Nanjing Biomedical Research Institute of Nanjing University).

### Statistical analysis

All data are expressed as the mean ± standard deviation (s.d.), and the number of samples is indicated in each figure legend. The statistical significance of differences was assessed using the Student’s t-test or Spearman correlation analysis. Results shown are representative of at least three independent experiments. Differences reached statistical significance with ^*^P < 0.05, ^**^P < 0.01 and ^***^P < 0.001. Statistical computations were performed using Prism software (Graph Pad).

## Result

### CPT inhibits the proliferation of melanoma cells

**Fig. 1:**
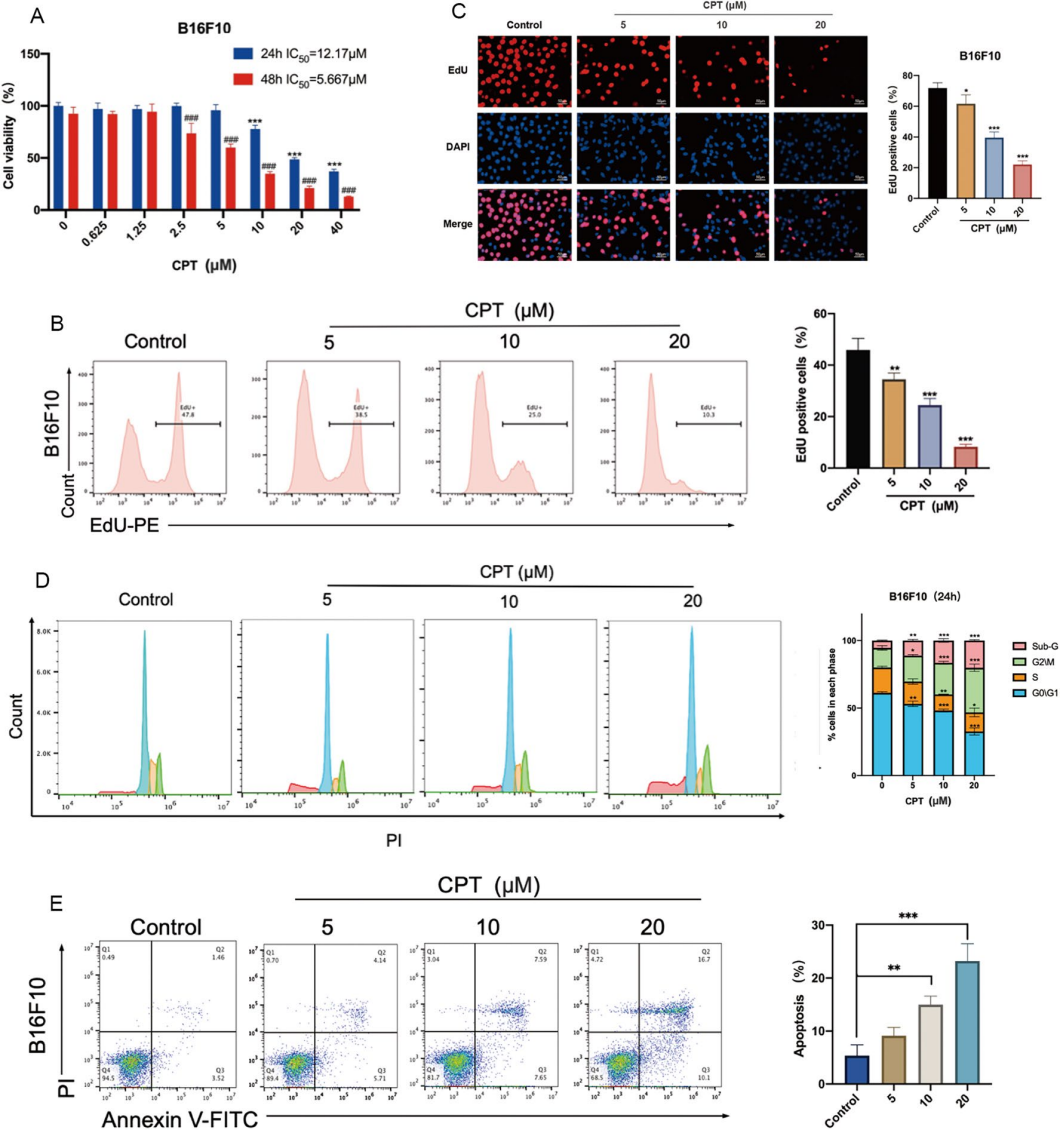
CPT inhibits cell proliferation and cell cycle of melanoma cells. **A** MTT assay for detecting the effect of CPT on the viability of B16F10 cells. **B** EDU staining detection of the effect of CPT on the proliferation of B16F10 cells and analysis by flow cytometry. **C** Fluorescence results of EDU staining detection of the effect of CPT on the proliferation of B16F10 cells. **D** Detection of the effect of CPT on cell cycle using propidium iodide (PI) staining and analysis by flow cytometry. **E** The effect of CPT on cell apoptosis. FITC labeled Annexin V was used to detect early apoptotic cells, while PI labeled necrotic cells or cells that lost cell membrane integrity in the late stage of apoptosis. Flow cytometry was used to analyze the cells. Compared with the control, * P < 0.05, ** P < 0.01, *** P < 0.001

We tested the effect of different concentrations of CPT on the proliferation of melanoma B16F10 (Fig. 1A) and A375 (Additional file 1: Figure S1A) cells by MTT assay. Based on the above results, we set the dosage of 0, 5, 10, and 20 μM for subsequent cell experiments. Later, we further used the EDU cell proliferation detection kit for direct detection of DNA synthesis in cells. The results showed that after 24 h of treatment with CPT on B16F10 (Fig. 1B, C) and A375 (Additional file 1: Figure S1B, C) cells, the EDU-positive cell rate in the treatment group was significantly reduced compared with the control.

The further results showed that in B16F10 cells, after 24 h of treatment with CPT, the proportion of G2/M phase cells obviously increased (Fig. 1D), and when the dosage of CPT exceeds 10 µM, it had a significant impact on cell apoptosis in B16F10 (Fig. 1E) and A375 cells (Additional file 1: Figure S1D). In conclusion, CPT inhibited proliferation by arresting cell cycle and inducing apoptosis in melanoma cells.

### CPT inhibits aerobic glycolysis in melanoma cells

**Fig. 2:**
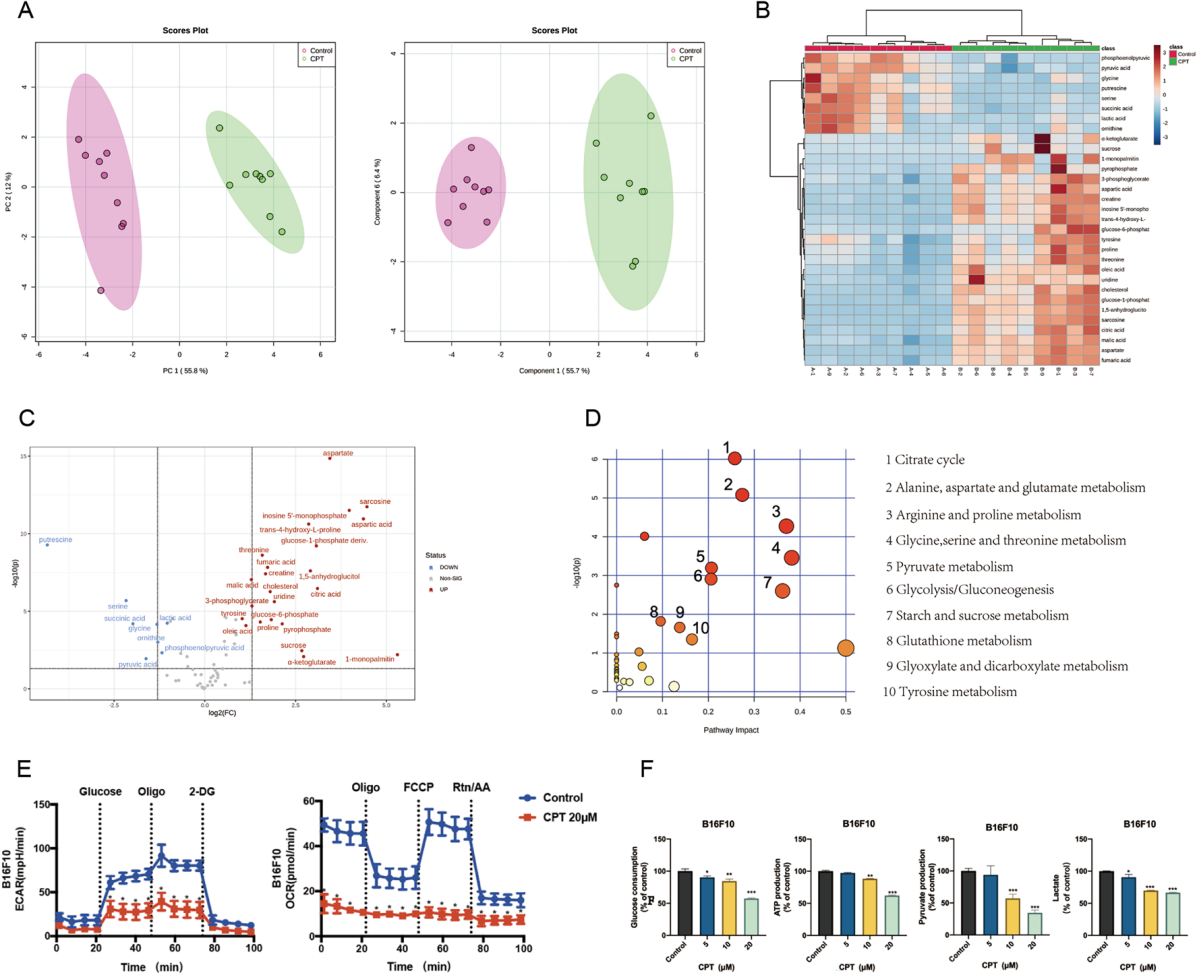
CPT inhibits glycolysis of melanoma cells. **A** PCA score map and PLS-DA score map of cell samples. **B** Metabolite heat map of significant changes in B16F10 cells after administration of CPT. **C** Differential metabolic product volcano map. **D** Topological map of differential metabolite signal pathways. **E** Effects of CPT on ECAR and OCR in B16F10 cells. **F** Effects of CPT on glucose uptake and production of lactate, pyruvate and ATP in melanoma cells. Compared with the control, * P < 0.05, ** P < 0.01, *** P < 0.001

The growth of tumor cells is closely related to metabolism. We used GC–MS to analyze endogenous metabolites in B16F10 cells treated with CPT. We found that the blank control group and the administration group can be clearly distinguished. This indicates a significant change in the endogenous metabolites of B16F10 cells after administration of CPT (Fig. 2A). A total of 31 significantly different metabolites were identified as potential biomarkers of CPT regulating the growth of melanoma cells (Fig. 2B, C, Additional file 6: Table S1). This suggested that CPT could inhibit the growth of melanoma cells by regulating some endogenous metabolites. The screened differential products were subjected to pathway enrichment analysis. The top 10 pathways with an impact value of > 0.1 and P < 0.05 are listed in Additional file 6: Table S2 and Fig. 2D. These results suggested that CPT may play a role in inhibiting the growth of melanoma cells mainly by affecting the glucose metabolism and amino acid metabolism of melanoma cells.

We used Seahorse XF instrument to monitor extracellular hydrogen ion concentration in real time. The results showed that CPT could significantly reduce the ECAR of B16F10 (Fig. 2E) and A375 cells (Additional file 2: Figure S2A). At the same time, CPT also reduced the basic oxygen consumption and maximum oxygen consumption of B16F10 (Fig. 2E) and A375 cells (Additional file 2: Figure S2A). Tumor cells consume a large amount of glucose, accompanied by ATP production, to meet the needs of rapidly proliferating tumor cells for energy and anabolic precursors. The experiment found that CPT decreased glucose consumption and pyruvate, lactate, and ATP production of B16F10 (Fig. 2F) and A375 (Additional file 2: Figure S2B) melanoma cells in a dose-dependent manner, confirming that CPT can block aerobic glycolysis of tumor cells.

### PFKFB3 mediates CPT inhibition of aerobic glycolysis

**Fig. 3:**
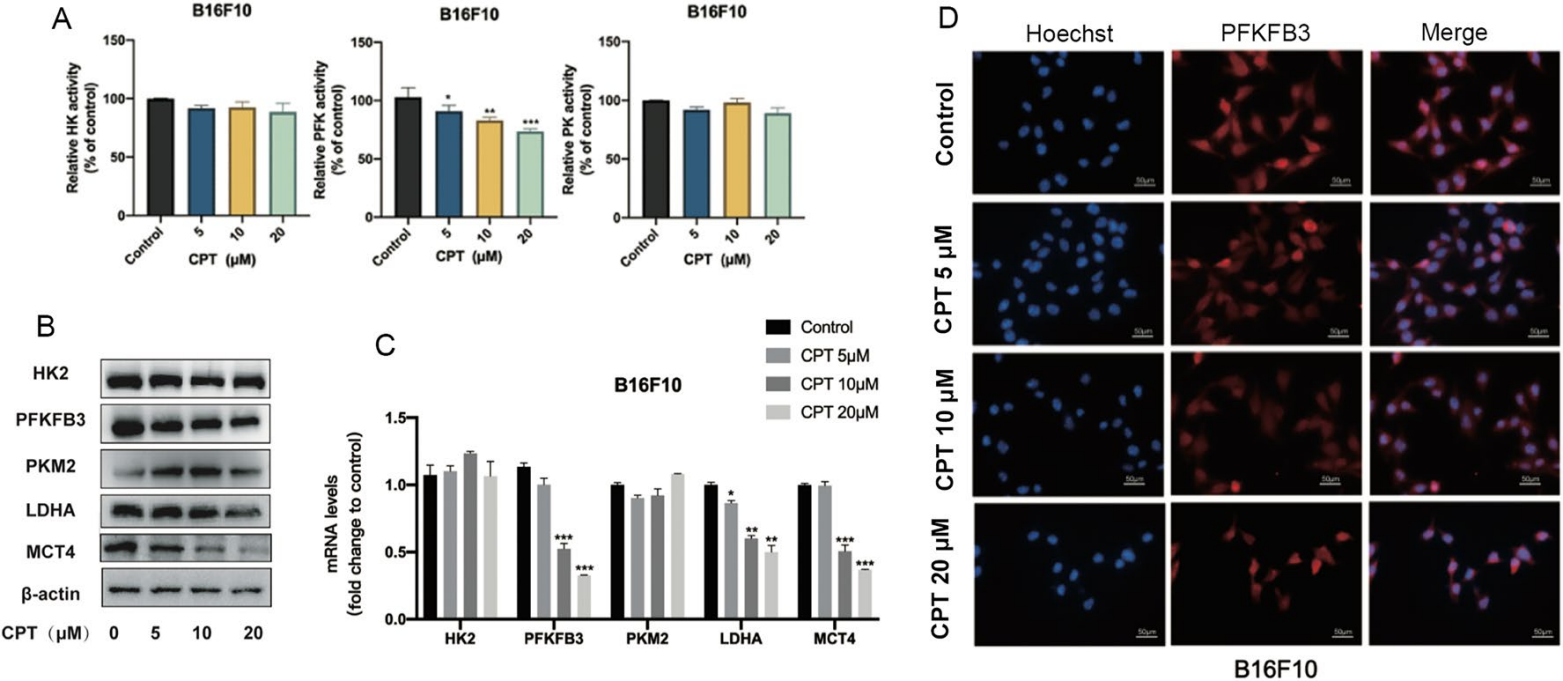
PFKFB3 mediates CPT inhibition of glycolysis of melanoma. **A** The activity of three key kinases of aerobic glycolysis was measured in B16F10 cells treated with CPT. **B** The glycolysis related proteins in melanoma cells were detected by western blot. **C** The level of glycolysis related proteins mRNA in melanoma cells was measured. **D** The expression of PFKFB3 was detected by fluorescence assay. Compared with the control, * P < 0.05, ** P < 0.01, *** P < 0.001

In the glycolysis process of tumor cells, there are three key kinases that regulate three irreversible steps. Using the enzyme activity detection kit to detect samples, we found that CPT had no significant effect on the hexokinase (HK) and pyruvate kinase (PK) activity of B16F10 cells and A375 cells, but had a significant and dose-dependent inhibitory effect on the phosphofructokinase (PFK) activity (Fig. 3A, Additional file 3: Figure S3A).

PFK is the second rate limiting enzyme in the glycolytic pathway. The results of western blot (Fig. 3B), qPCR (Fig. 3C) and immunofluorescence (Fig. 3D) showed that the expression and transcription level of PFKFB3 was inhibited significantly, as well as the downstream lactate dehydrogenase A (LDHA) and monocarboxylate transporter 4 (MCT4). It suggested that CPT inhibited the conversion of fructose-6-phosphate to fructose-1,6-biphosphate during the course of aerobic glycolysis in cancer cells, finally leading to decreasing pyruvate production, preventing lactate from transporting out of the cell membrane and blocking the glucose uptake by the way of negative feedback.

### Four prognostic biomarkers for melanoma-related glycolytic genes were predicted

**Fig. 4:**
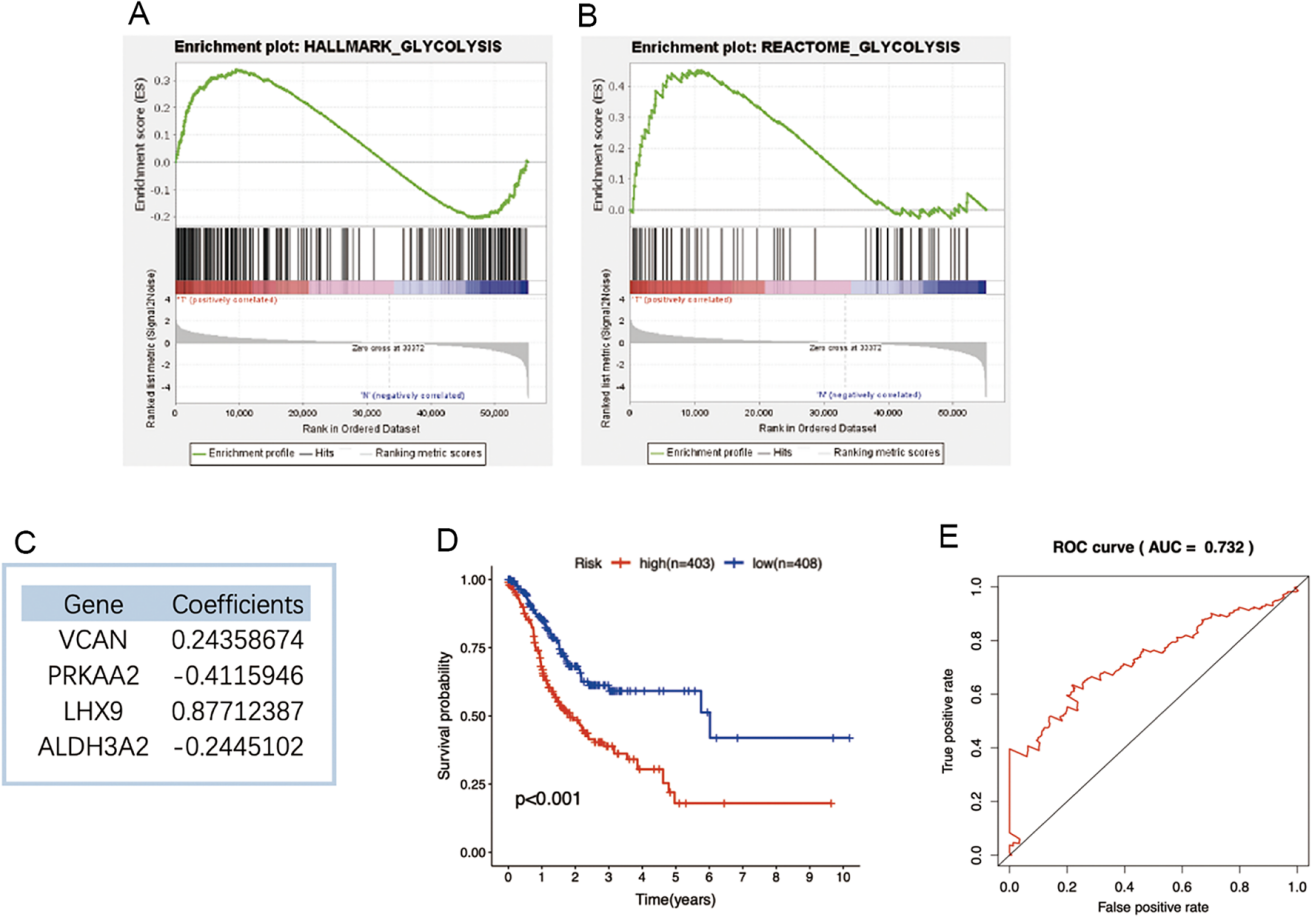
Four prognostic biomarkers for melanoma-related glycolytic genes were predicted. **A**, **B** Enrichment results of glycolytic preset genes in GSEA. **C** Four prognostic biomarkers for melanoma-related glycolytic genes were predicted. **D** Regression coefficient of glycolysis prognosis model and survival curve of melanoma patients in high and low risk groups. **E** ROC curve for assessing prognosis

In order to further explore its internal mechanism, we downloaded the transcriptome data of 471 melanoma tissues and 340 normal tissues from TCGA and GTEX databases for comparing and analyzing. Detailed operating procedures can be found in Additional file 7. The results showed that the peak value of the enrichment curve appeared above the tumor group, which also proved that glycolysis was more active in melanoma than in normal tissues (Fig. 4A, B). Based on the enrichment results of GSEA, we extracted the expression levels of glycolytic related genes in each sample. We conducted differential analysis filtering between normal and tumor samples and identified 13 glycolytic genes related to prognosis. It contains 4 low-risk genes and 9 high-risk genes (Additional file 6: Table S3). Based on the prognosis related glycolysis genes, COX regression analysis was carried out and optimized to obtain four prognostic markers of melanoma (Fig. 4C). And the risk value of patients was predicted, the survival curve results are shown in Fig. 4D. The value of the area under the ROC curve as 0.732 (Fig. 4E) is more than 0.7, suggesting that the model has good accuracy in predicting the survival of melanoma patients. Among four prognostic markers of melanoma, PRKAA2 gene is negatively correlated to the energy metabolism because the protein encoded by the PRKAA2 gene is a catalytic subunit of AMPK [18]. Thus, we hypothesized that AMPK maybe mediate CPT inhibition of glycolysis of melanoma.

### CPT inhibits HIF-1α by activating AMPK and promotes the death of melanoma cells

**Fig. 5:**
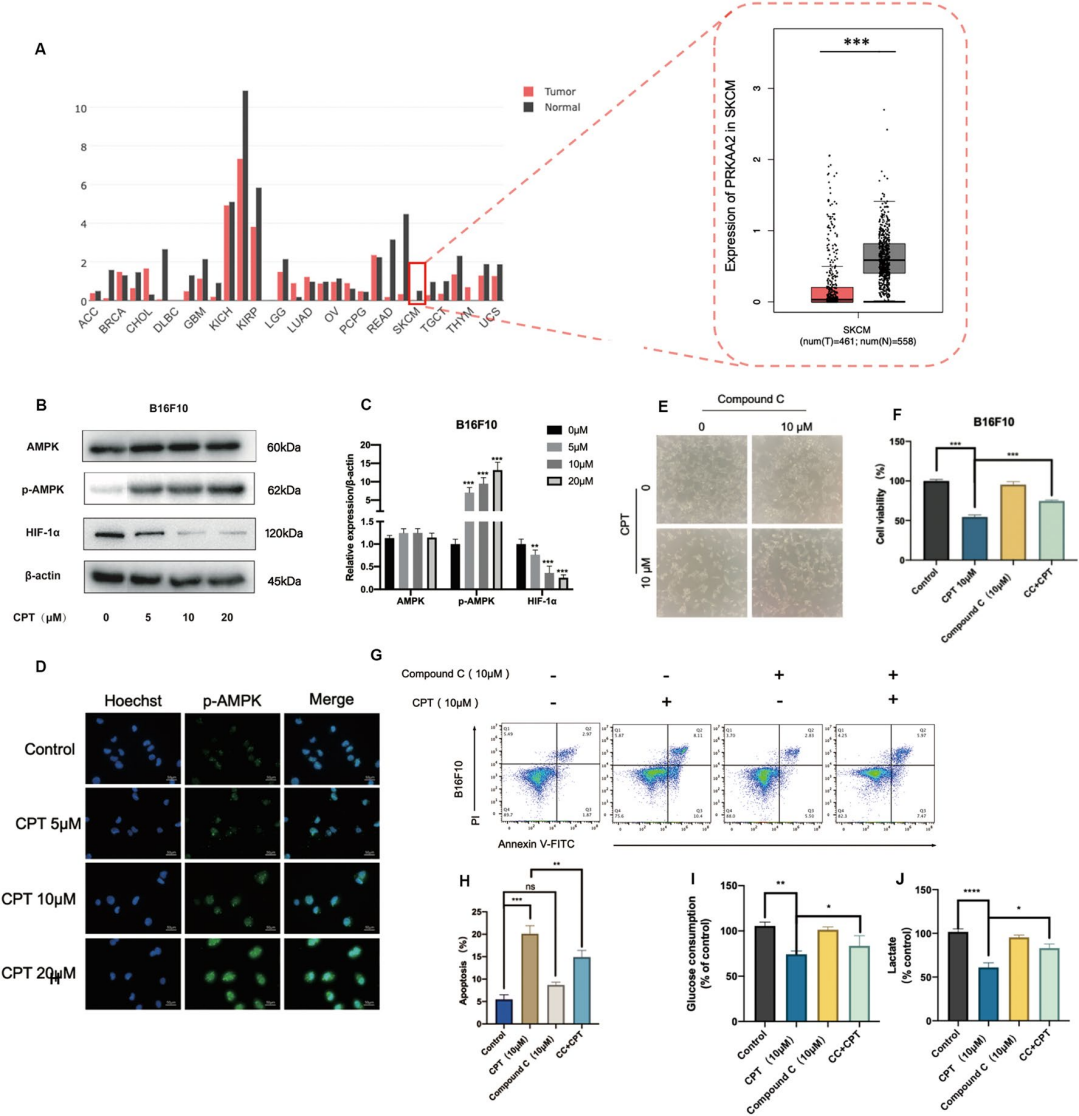
AMPK mediates CPT inhibition of glycolysis in melanoma cells. **A** Expression of PRKAA2 gene in different tumors in GEPIA database (^***^ P < 0.001). **B** Western blot was used to detect the effect of CPT on the expression of AMPK, p-AMPK and HIF-1α in B16F10 melanoma cells. **C** Quantitative results of AMPK, p-AMPK, HIF-1α. **D** Effect of CPT on p-AMPK expression in melanoma cells detected by cellular immunofluorescence. The effect of CPT combined with AMPK inhibitor compound C on B16F10 cells: **E** the morphology of melanoma cells under inverted microscopy. **F** MTT detection of the viability of melanoma cells. **G**, **H** Detection of apoptosis of melanoma cells by Flow cytometry. **I**, **J** The glucose consumption and lactate production. Compared with the control, * P < 0.05, ** P < 0.01, *** P < 0.001

To further confirm the hypothesis, we firstly investigated the expression of PRKAA2 gene in different tumors through the GEPIA database. It was found that the expression of PRKAA2 gene was significantly lower in tumor tissues of patients with melanoma than in normal tissues (Fig. 5A). Then we found that CPT could activate AMPK as the phosphorylation of AMPK increases by a concentration-dependent way (Fig. 5B, C) (Additional file 4: Figure S4A, B). AMPK is a highly conserved cellular energy state sensor in eukaryotes, used to maintain intracellular energy homeostasis [19]. Inhibiting the activity of AMPK increases the glycolytic metabolism of tumor cells and promotes the growth of lymphoma, and silencing HIF-1α can reverse this metabolic transformation and reduce the proliferation advantage of tumor cells caused by AMPK inactivation [20]. We also found that CPT significantly inhibited the expression of HIF-1α (Fig. 5B, C, Additional file 4: Figure S4A, B). In addition, cellular immunofluorescence detection of p-AMPK expression also showed the same trend (Fig. 5D, Additional file 4: Figure S4C). Therefore, we hypothesized that CPT acted on AMPK/HIF-1α signal pathway to regulate the glycolysis of tumor cells.

Afterwards, we added AMPK inhibitor compound C for further validation. Compared with the 10 μM CPT group, the addition of Compound C weakened the inhibitory effect on B16F10 cells (Fig. 5E, F). The results of the cell apoptosis test kit showed that compound C weakened the apoptosis induced by CPT (Fig. 5G, H, Additional file 4: Figure S4D, E). Additionally, The data of the glucose and lactate detection showed that Compound C had no significant effect on glucose uptake and lactate secretion in B16F10 cells, while the combination group significantly increased the glucose uptake and lactate secretion of B16F10 cells (Fig. 5I, J). These results indicated that CPT inhibiting glycolysis of melanoma cells depended on the activation of AMPK.

### Silencing AMPK can partially reverse CPT’s inhibitory effect on cell proliferation, apoptosis and glycolysis

**Fig. 6:**
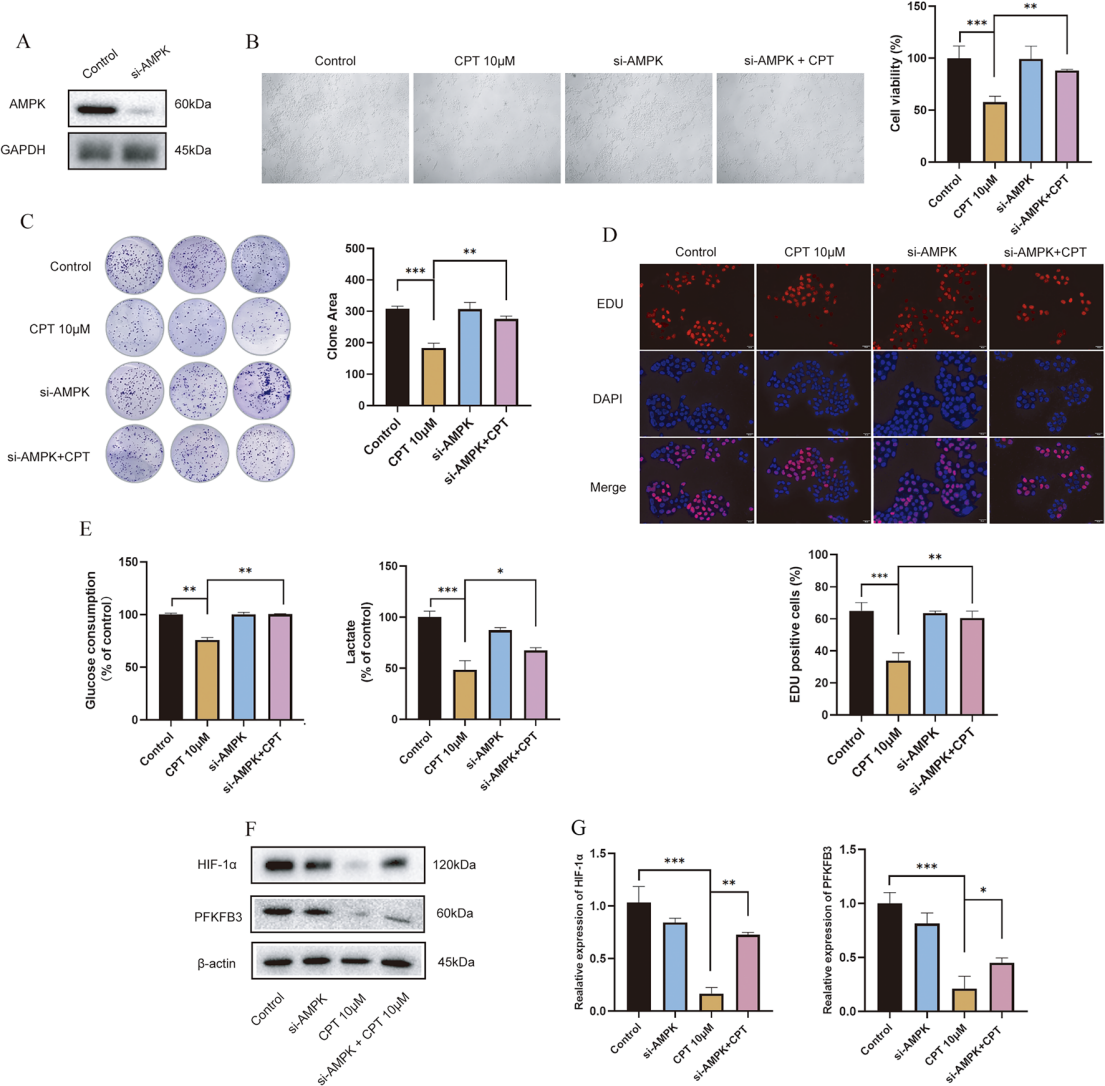
Knockdowning AMPK partially reverses CPT’s effect on melanoma cells in vitro. Si-AMPK mimics were used to knockdown the AMPK expression in A375 cells, then the cells were treated with CPT for 24 h followed with the following assays. **A** Western blot for the expression of AMPK in A375 cells. **B** CCK assay for detecting the effect of CPT on the viability of A375 cells. **C** Representative images and quantitative analysis was conducted on the formation of cell clones by colony-formation assay. **D** Fluorescence results of EDU staining on the proliferation of A375 cells. **E** Detection of glycolytic metabolites of A375 cells. **F**, **G** Western blot for HIF-1α and PFKFB3 and semiquantitative grayscale analysis. Compared with 10 μM CPT, * P < 0.05, ** P < 0.01, *** P < 0.001

To further demonstrate that CPT’s inhibition of melanoma cells is related to activation of AMPK, we used si-RNA mimics to knockdown AMPK in A375 cells to compare the difference of inhibitory effect of CPT on melanoma cells with different expression of AMPK. As shown in Fig. 6A, AMPK was successfully silenced in A375 cells. Cell viability of 10 μM CPT treatment in A375 cells is significantly less than that in AMPK-silencing A375 cells (Fig. 6B), suggesting that silencing AMPK can partially reverse CPT’s inhibitory effect. And the clone assay and EDU assay gave the same results as cell viability (Fig. 6C, D). Under treatment of CPT, glucose consumption and lactate production of AMPK-silencing cells are more (Fig. 6E). Expression of HIF-1α and PFKFB3 inhibited in A375 cells were obviously recovered in AMPK-silencing A375 cells (Fig. 6F, G). All above suggested that activation of AMPK mediated CPT inhibition of aerobic glycolysis leading to cell death.

### CPT inhibits the growth of melanoma in vivo

**Fig. 7:**
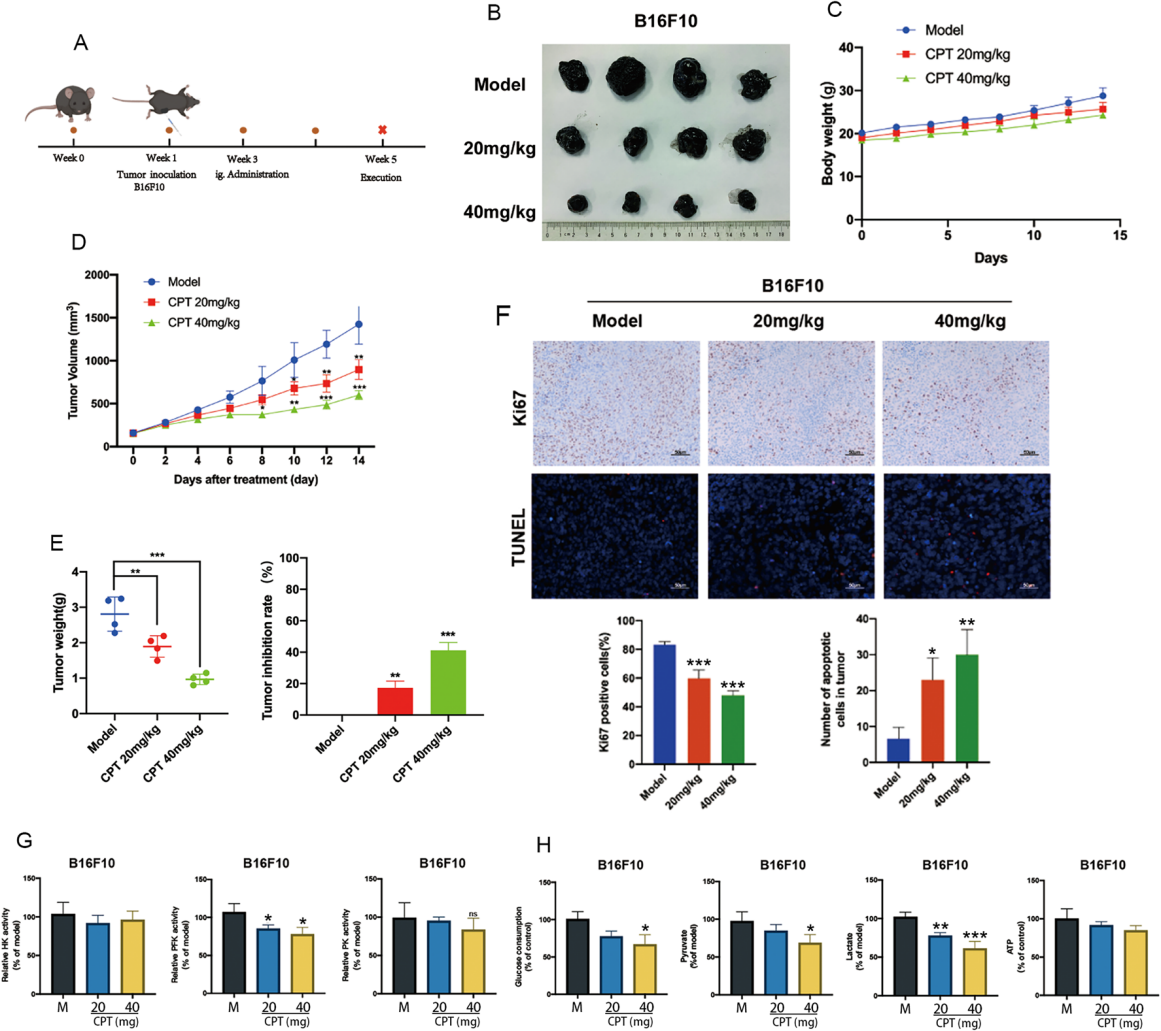
CPT inhibits the growth of melanoma in vivo. **A** Schematic diagram for the construction of subcutaneous transplantation tumor models with B16F10 cells. **B** The representive image of melanoma solid tumors dissected from the mice transplanted with B16F10. **C** Body weight changes in mice. **D** Measurement of tumor volume in mice using a vernier caliper. **E** Tumor weight and tumor growth inhibition rate. **F** Ki67 and TUNEL staining of B16F10 cell tumor tissues. **G** The key kinases activity of glycolysis was measured in melanoma tissue. **H** The glycolytic metabolites were detedted in melanoma tissue. Compared with the model group, * P < 0.05, ** P < 0.01, *** P < 0.001

We evaluated the anti-tumor effect of CPT in vivo by establishing tumor graft models of melanoma in B16F10 and A375 cells (Fig. 7A, Additional file 5: Figure S5A). In the tumor model, compared with the model group, the tumor weight and volume of the CPT treatment group were significantly smaller though the body weight was not affected (Fig. 7B–E, Additional file 5: Figure S5B-E). All these results indicated that CPT had a certain inhibitory effect on the growth of melanoma in vivo. Tumor tissue was stained with Ki67 and TUNEL. Compared with the model group, the administration of 20 mg/kg and 40 mg/kg CPT significantly reduced the number of Ki67-positive cells in tumor tissue, while the number of TUNEL stained positive cells significantly increased (Fig. 7F, Additional file 5: Figure S5F). This indicated that CPT could inhibit cell proliferation and promote cell apoptosis in B16F10 and A375 cells transplanted tumor tissues. Immunohistochemical method was used to detect the expression levels of p-AMPK and apoptosis related proteins, Bax and Bcl-2, in A375 cells transplanted tumor tissues. CPT increased the level of AMPK phosphorylation in tumor tissue and significantly reduced the expression of pro-apoptotic protein Bcl-2 in tumor tissue, while the expression of anti-apoptotic protein Bax significantly increased (Additional file 5: Figure S5G).

We further detected the activity of glycolytic kinase within tumor tissue and found that CPT just inhibited PFK kinase activity (Fig. 7G, Additional file 5: Figure S5H). By detecting glycolytic metabolites, we found that compared with the model group, CPT significantly inhibited internal glucose consumption and pyruvate production, as well as lactate secretion in subcutaneous transplanted tumor tissues (Fig. 7H, Additional file 5: Figure S5I). Taken together, CPT inhibited melanoma growth in vivo through AMPK-mediated glycolysis.

## Discussion

Highly-changed energy metabolism is an important characteristic of cancer cells that represents one of the “hallmarks of cancer” [21]. Glycolysis is an important mode of metabolism in cancer cells and plays an important role in cancer progression. High throughput glycolysis provides not only rapid energy supply, but also raw materials for the synthesis of various biological macromolecules in cancer cells, as the various intermediate metabolites produced during glycolysis are essential precursors for various other metabolic pathways [22]. A large amount of pyruvate is converted into lactic acid without entering the tricarboxylic acid cycle to complete oxidative phosphorylation, leading to reducing the electron transfer of the mitochondrial respiratory chain and the production of reactive oxygen species. However, maintaining a medium level of ROS for a long time is still conducive to tumor progress [12]. Thus, interrupting or possibly disrupting tumor glycolysis will impact tumor growth by energy depletion as well as sensitization to therapeutics especially, in light of the recent reports that have elucidated cancer-specific advantages of aerobic glycolysis [23].

Melanoma is a skin cancer caused by a malignancy of melanocytes. Incidence of melanoma is rapidly increasing worldwide, resulting in public health problems and burden [24]. Hypoxia is a typical characteristic of solid tumors, including melanoma. It regulates cancer cell metabolism reprogramming, promotes tumor cell growth, invasion, and infiltration. HIF-1α is the main transcription factor under hypoxia and mediates glycolysis by regulating the expression of glycolytic genes such as GLUT1, HK, PKM [25, 26]. AMPK regulates metabolism in response to the cellular energy states. Under energy stress, AMP stabilizes the active AMPK conformation, in which the kinase activation loop (AL) is protected from protein phosphatases, thus keeping the AL in its active, phosphorylated state [27]. AMPK is a negative regulator of aerobic glycolysis and inhibits tumor growth when activated [15]. What is more, HIF-1α is a key mediator of AMPK-dependent effects on cellular metabolism [15]. Inactivation of AMPK promotes a metabolic shift to aerobic glycolysis, increased allocation of glucose carbon into lipids, and biomass accumulation. These metabolic effects require normoxic stabilization of HIF-1α, as silencing HIF-1α reverses the shift to aerobic glycolysis and the biosynthetic and proliferative advantages conferred by reduced AMPK signaling [15]. In short, AMPK/HIF-1α signaling pathway is a key pathway for maintaining cellular oxidative phosphate and glycolysis balance.

**Fig. 8:**
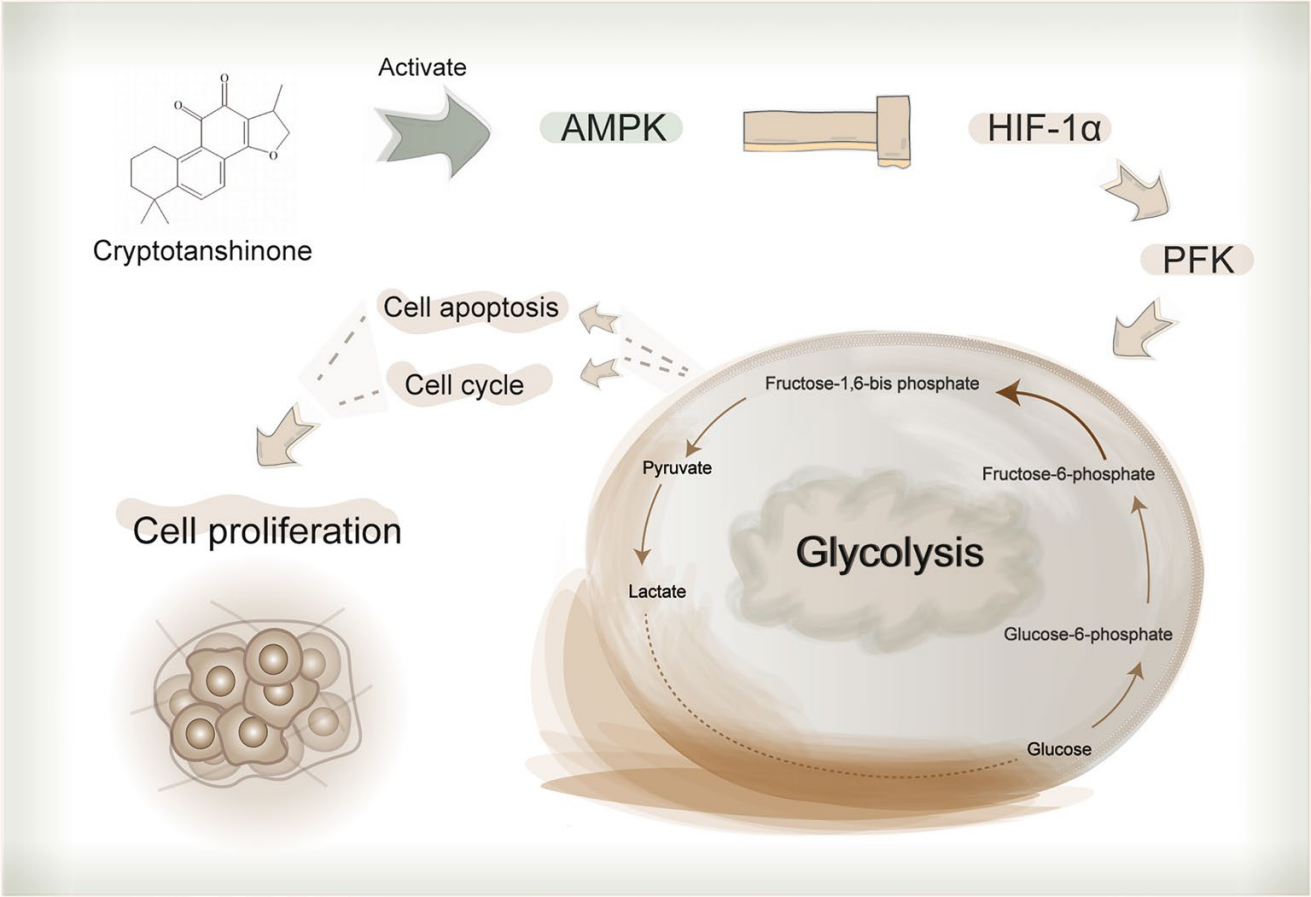
The molecular mechanism diagram of CPT inhibiting melanoma cell proliferation

We have made much attempt to elucidate the molecular mechanism of CPT against cancer. CPT can inhibit cancer cell proliferation by blocking mTOR-mediated cyclin D1 and Rb phosphorylation [28]. However, CPT can not bind to FKBP12, but activate AMPK-TSC2 axis to inhibit mTORC1 signaling [16]. Furthermore, we also found that CPT inhibited mTOR dependent on estrogen receptor ɑ in breast cancer [29], and reversed multidrug resistance of breast cancer by inhibiting BCRP oligomer formation [30]. Here, CPT reduced the content of key glycolytic metabolites, inhibited the kinase of PFK and the protein expression of PFKFB3 in melanoma cells, suggesting that CPT can interrupt the glucose metabolism of melanoma cells. PFK increases glycolytic flux and lactate production, promoting cell cycle progression through cyclin dependent kinases [31–33]. Inhibition of PFKFB3 inhibited cell proliferation, arrested cell cycle in G0/G1 phase by suppressing the Cyclin-CDKs/Rb signaling pathway [33]. Thus, we thought that PFK mediated CPT inhibition of mTOR-mediated cyclin D1 and Rb phosphorylation. Inhibiting PFKFB3 also induced cell apoptosis through mitochondria by decreasing the ratio of Bcl-2/Bax in cancer cells [33]. This further clued that PFKFB3 was necessary for CPT inducing melanoma cell apoptosis. Furthermore, overexpression of PFKFB3 under hypoxic condition is correlated with enhanced level of HIF-1α protein in gastric cancer cells [34]. And HIF-1α is positively controlled by the upstream mTOR to activate the transcription of numerous target genes for adapting the hypoxic environment [35]. Therefore, our results supported that CPT activated AMPK but inhibited mTOR-mediated HIF-1α expression. Then the glycolytic speed-limiting kinase PFK was inhibited and the glucose uptake and lactate secretion of melanoma cells were decreased, resulting in cell cycle arrested and apoptosis (Fig. 8).

## Conclusions

CPT activated AMPK, inhibited the expression of HIF-1α and blocked the PFK-controlled glycolysis, leading to melanoma cell death. CPT indicated an obvious inhibition on melanoma and should be a promising agent for melanoma therapy.

### Supplementary Information

**Additional file 1: Figure S1.** CPT inhibits cell proliferation and induces apoptosis in A375 melanoma cells. (A) MTT assay for detecting the effect of CPT on the viability of A375 cells. (B) EDU staining detection of the effect of CPT on the proliferation of A375 cells and analysis by flow cytometry. (C) Fluorescence results of EDU staining detection of the effect of CPT on the proliferation of A375 cells. (D) The effect of CPT on cell apoptosis. FITC labeled Annexin V was used to detect early apoptotic cells, while PI labeled necrotic cells or cells that lost cell membrane integrity in the late stage of apoptosis. Flow cytometry was used to analyze the cells. Compared with the control, * P < 0.05, ** P < 0.01, *** P < 0.001.

**Additional file 2: Figure S2.** CPT inhibits glycolysis of A375 melanoma cells. (A) Effects of CPT on ECAR and OCR in A375 cells. (B) Effects of CPT on glucose uptake and production of lactate, pyruvate and ATP in A375 melanoma cells. Compared with the control, * P < 0.05, ** P < 0.01, *** P < 0.001.

**Additional file 3: Figure S3.** PFKFB3 mediates CPT inhibition of glycolysis of melanoma. (A) The activity of three key kinases of aerobic glycolysis was measured in A375 cells treated with CPT. (B) The expression of PFKFB3 was detected by fluorescence assay. Compared with the control, * P < 0.05, ** P < 0.01, *** P < 0.001.

**Additional file 4: Figure S4.** AMPK mediates CPT inhibition of glycolysis in A375 melanoma cells. (A) Western blot was used to detect the effect of CPT on the expression of AMPK, p-AMPK and HIF-1α in B16F10 melanoma cells. (B) Quantitative results of AMPK, p-AMPK, HIF-1α. (C) Effect of CPT on p-AMPK expression in melanoma cells detected by cellular immunofluorescence. The effect of CPT combined with AMPK inhibitor compound C on A375 cells: (D) Detection of apoptosis of melanoma cells by flow cytometry. (E) The quantitative analysis of Flow cytometry data. Compared with the control, * P < 0.05, ** P < 0.01, *** P < 0.001.

**Additional file 5: Figure S5.** CPT inhibits the growth of melanoma in vivo. (A) Schematic diagram for the construction of subcutaneous transplantation tumor models with A375 cells. (B) The representative image of melanoma solid tumors dissected from the mice transplanted with A375 cells. (C) Body weight changes in mice. (D) Measurement of tumor volume in mice using a vernier caliper. (E) Tumor weight and tumor growth inhibition rate. (F) Ki67 and TUNEL staining of A375 cell tumor tissues. (G) Immunohistochemical analysis of p-AMPK, Bcl2 and Bax. (H) The key kinases activity of glycolysis was measured in melanoma tissue. (I) The glycolytic metabolites were detedted in melanoma tissue. Compared with the model group, * P < 0.05, ** P < 0.01, *** P < 0.001.

**Additional file 6: Table S1.** Differential metabolites in cell samples. **Table S2.** Pathway analysis of differential metabolites. **Table S3.** Glycolysis genes related to prognosis in patients with melanoma.

**Additional file 7. **Additional Methods.

## Data Availability

The datasets supporting the conclusions of this article are included within the article and its additional files.

## Abbreviations

HIF-1α: Hypoxia inducible factor 1α
AMPK: Adenosine monophosphate-activated protein kinase
ATP: Adenosine triphosphate
AL: Activation loop
CPT: Cryptotanshinone
PBS: Phosphate-buffered saline
HRP: Horseradish peroxidase
PFK: Fructose phosphate kinase
MCT4: Monocarboxylate transporter 4
GLUT1: Glucose transporter 1
CDKs: Cyclin dependent kinases
HREs: Hypoxia response elements
TCM: Traditional Chinese Medicine
LDHA: Lactate dehydrogenase A
ECAR: Extracellular acidification rate
OCR: Oxygen consumption rate
BCRP: Breast cancer resistance protein
