# Supplementary figures and images for: Cryptotanshinone inhibits PFK-mediated aerobic glycolysis by activating AMPK pathway leading to blockade of cutaneous melanoma
Source: Chin Med. 2024 Mar 7;19:45. doi: 10.1186/s13020-024-00913-1 (PMC10921599; doi:10.1186/s13020-024-00913-1)

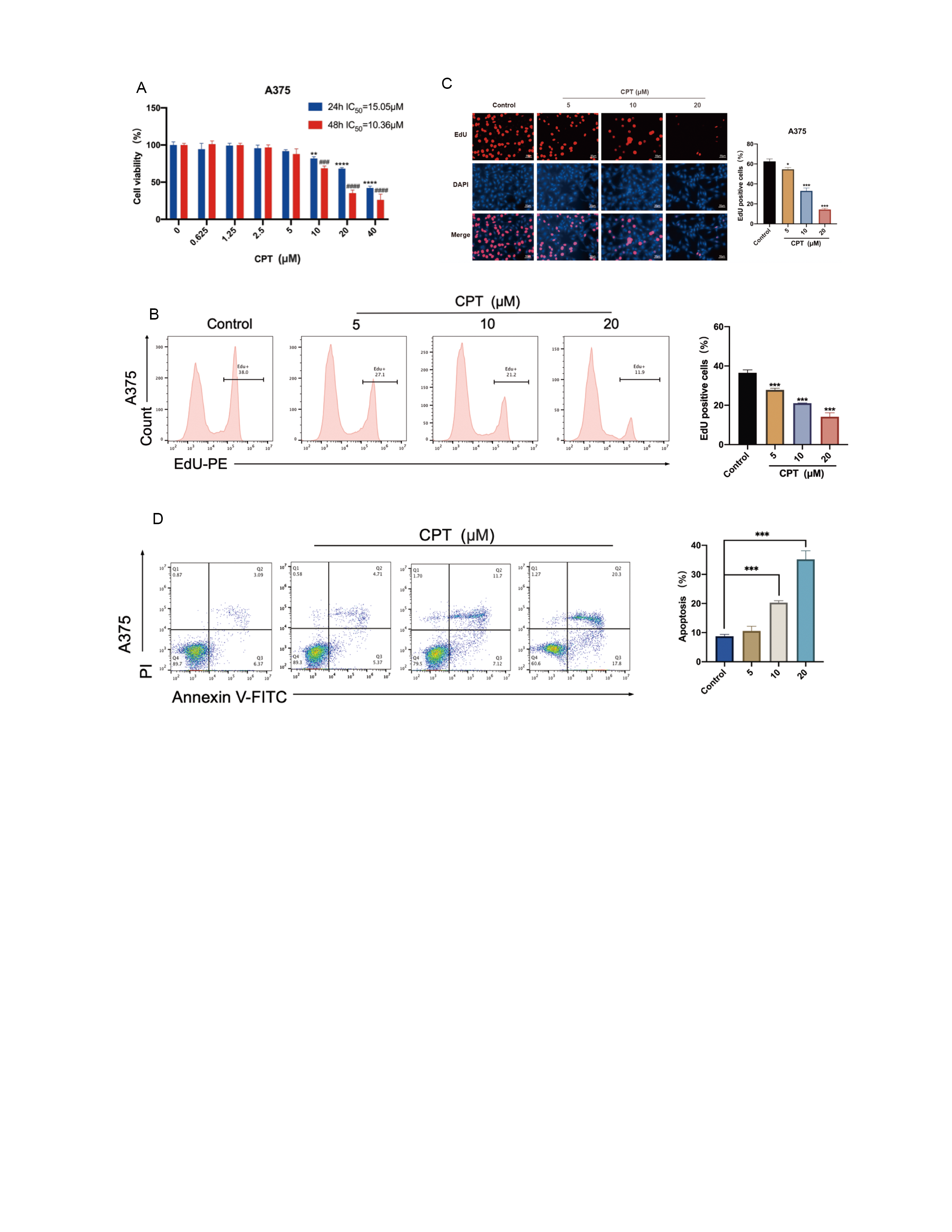

Supplement: Supplementary file 1 — Additional file 1: Figure S1. CPT inhibits cell proliferation and induces apoptosis in A375 melanoma cells. (A) MTT assay for detecting the effect of CPT on the viability of A375 cells. (B) EDU staining detection of the effect of CPT on the proliferation of A375 cells and analysis by flow cytometry. (C) Fluorescence results of EDU staining detection of the effect of CPT on the proliferation of A375 cells. (D) The effect of CPT on cell apoptosis. FITC labeled Annexin V was used to detect early apoptotic cells, while PI labeled necrotic cells or cells that lost cell membrane integrity in the late stage of apoptosis. Flow cytometry was used to analyze the cells. Compared with the control, * P < 0.05, ** P < 0.01, *** P < 0.001. [file 13020_2024_913_MOESM1_ESM.tif]

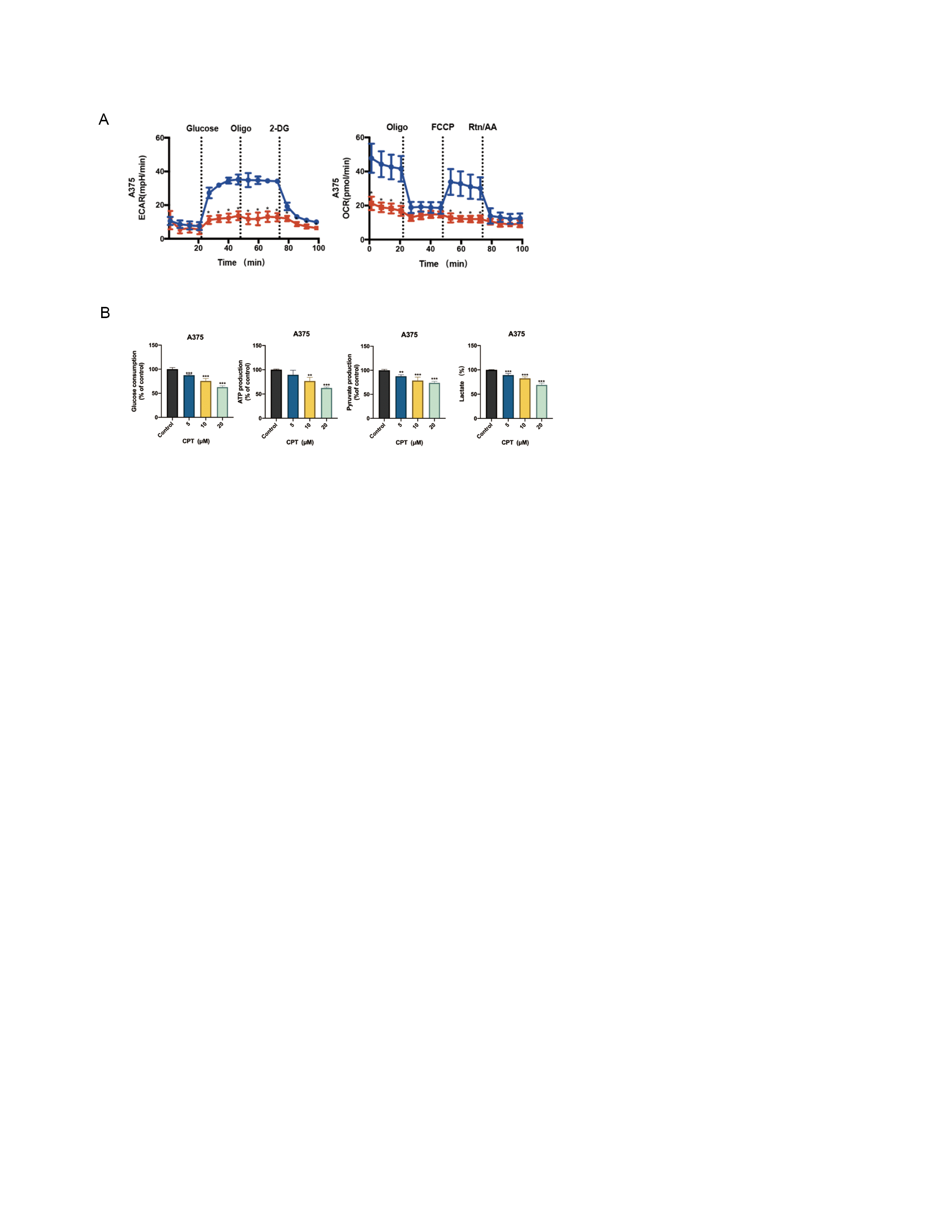

Supplement: Supplementary file 2 — Additional file 2: Figure S2. CPT inhibits glycolysis of A375 melanoma cells. (A) Effects of CPT on ECAR and OCR in A375 cells. (B) Effects of CPT on glucose uptake and production of lactate, pyruvate and ATP in A375 melanoma cells. Compared with the control, * P < 0.05, ** P < 0.01, *** P < 0.001. [file 13020_2024_913_MOESM2_ESM.tif]

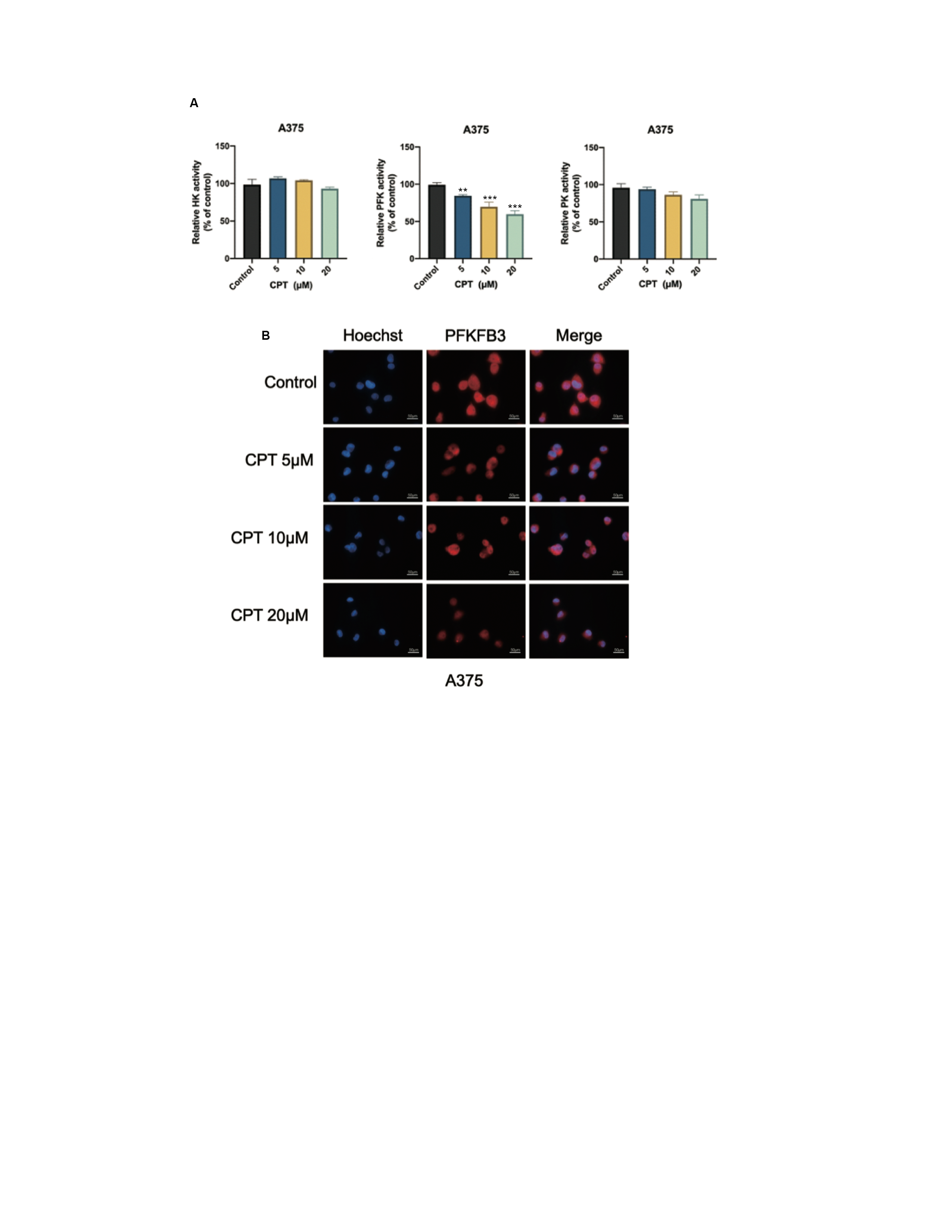

Supplement: Supplementary file 3 — Additional file 3: Figure S3. PFKFB3 mediates CPT inhibition of glycolysis of melanoma. (A) The activity of three key kinases of aerobic glycolysis was measured in A375 cells treated with CPT. (B) The expression of PFKFB3 was detected by fluorescence assay. Compared with the control, * P < 0.05, ** P < 0.01, *** P < 0.001. [file 13020_2024_913_MOESM3_ESM.tif]

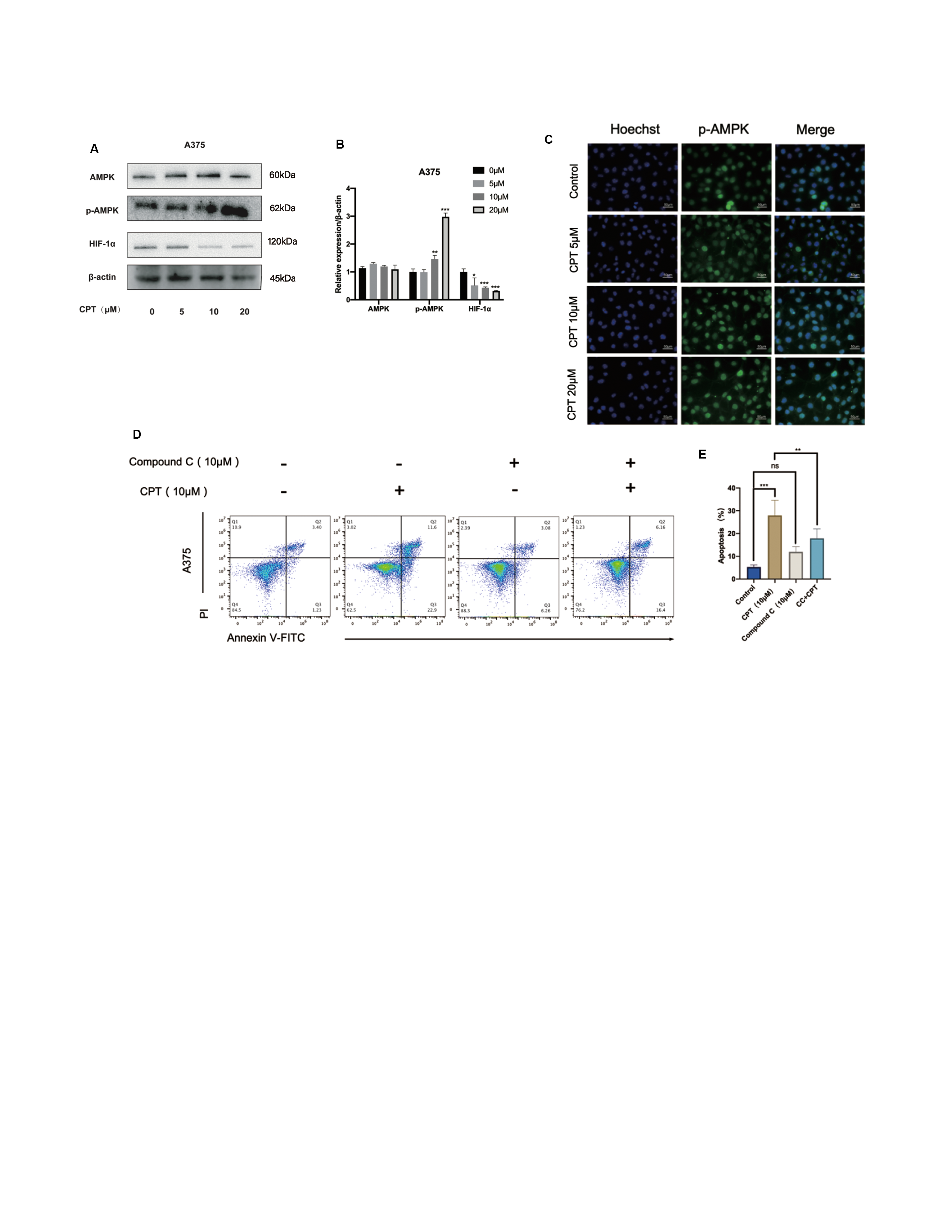

Supplement: Supplementary file 4 — Additional file 4: Figure S4. AMPK mediates CPT inhibition of glycolysis in A375 melanoma cells. (A) Western blot was used to detect the effect of CPT on the expression of AMPK, p-AMPK and HIF-1α in B16F10 melanoma cells. (B) Quantitative results of AMPK, p-AMPK, HIF-1α. (C) Effect of CPT on p-AMPK expression in melanoma cells detected by cellular immunofluorescence. The effect of CPT combined with AMPK inhibitor compound C on A375 cells: (D) Detection of apoptosis of melanoma cells by flow cytometry. (E) The quantitative analysis of Flow cytometry data. Compared with the control, * P < 0.05, ** P < 0.01, *** P < 0.001. [file 13020_2024_913_MOESM4_ESM.tif]

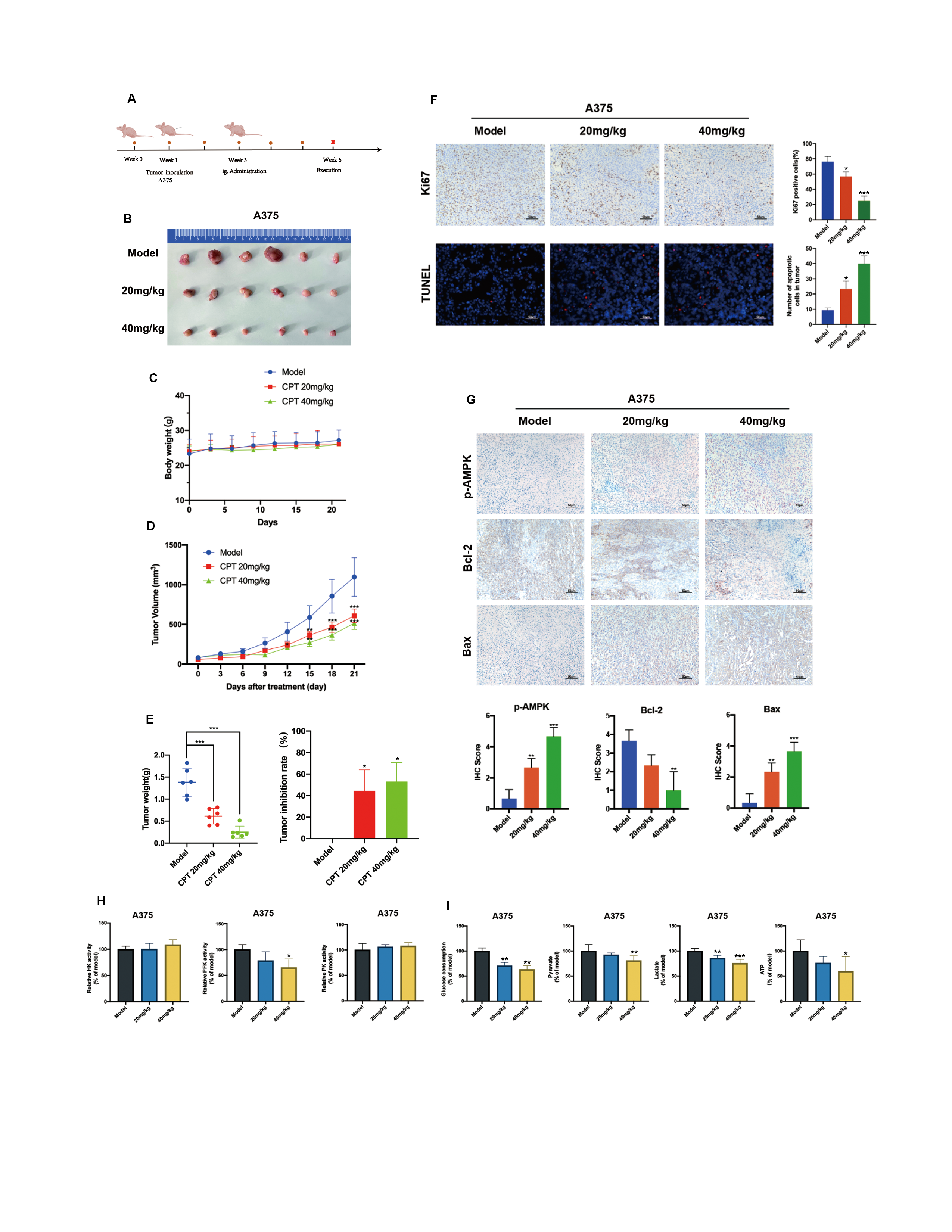

Supplement: Supplementary file 5 — Additional file 5: Figure S5. CPT inhibits the growth of melanoma in vivo. (A) Schematic diagram for the construction of subcutaneous transplantation tumor models with A375 cells. (B) The representative image of melanoma solid tumors dissected from the mice transplanted with A375 cells. (C) Body weight changes in mice. (D) Measurement of tumor volume in mice using a vernier caliper. (E) Tumor weight and tumor growth inhibition rate. (F) Ki67 and TUNEL staining of A375 cell tumor tissues. (G) Immunohistochemical analysis of p-AMPK, Bcl2 and Bax. (H) The key kinases activity of glycolysis was measured in melanoma tissue. (I) The glycolytic metabolites were detedted in melanoma tissue. Compared with the model group, * P < 0.05, ** P < 0.01, *** P < 0.001. [file 13020_2024_913_MOESM5_ESM.tif]
