# Supplementary material for: Cryptotanshinone inhibits PFK-mediated aerobic glycolysis by activating AMPK pathway leading to blockade of cutaneous melanoma
Source: Chin Med. 2024 Mar 7;19:45. doi: 10.1186/s13020-024-00913-1 (PMC10921599; doi:10.1186/s13020-024-00913-1)
Supplement: Supplementary file 6 — Additional file 6: Table S1. Differential metabolites in cell samples. Table S2. Pathway analysis of differential metabolites. Table S3. Glycolysis genes related to prognosis in patients with melanoma. [file 13020_2024_913_MOESM6_ESM.doc]

**Table S**1

| **Table S1. Differential metabolites in cell samples** | | | | |
| --- | --- | --- | --- | --- |
| No. | Metabolite | Administration group vs control group | | |
|  |  | Fold Change | P value | Trend |
| 1 | putrescine | 0.04942 | 5.28×10^-10^ | ↓*** |
| 2 | serine | 0.2223 | 2.05×10^-6^ | ↓*** |
| 3 | succinic acid | 0.2533 | 6.33×10^-5^ | ↓*** |
| 4 | pyruvic acid | 0.3254 | 1.13×10^-2^ | ↓* |
| 5 | glycine | 0.4008 | 6.94×10^-5^ | ↓*** |
| 6 | ornithine | 0.4072 | 9.82×10^-4^ | ↓*** |
| 7 | phosphoenolpyruvic acid | 0.4409 | 4.69×10^-3^ | ↓** |
| 8 | lactic acid | 0.4878 | 5.79×10^-5^ | ↓*** |
| 9 | tyrosine | 2.039 | 2.99×10^-5^ | ↑*** |
| 10 | oleic acid | 2.191 | 8.38×10^-5^ | ↑*** |
| 11 | malic acid | 2.431 | 9.10×10^-8^ | ↑*** |
| 12 | 3-phosphoglycerate | 2.457 | 4.53×10^-6^ | ↑*** |
| 13 | proline | 2.88 | 4.90×10^-5^ | ↑*** |
| 14 | threonine | 2.99 | 2.42×10^-9^ | ↑*** |
| 15 | creatine | 3.182 | 3.85×10^-8^ | ↑*** |
| 16 | fumaric acid | 3.31 | 1.48×10^-8^ | ↑*** |
| 17 | cholesterol | 3.479 | 5.40×10^-7^ | ↑*** |
| 18 | glucose-6-phosphate | 3.553 | 3.46×10^-5^ | ↑*** |
| 19 | uridine | 3.767 | 2.43×10^-6^ | ↑*** |
| 20 | pyrophosphate | 4.375 | 6.48×10^-5^ | ↑*** |
| 21 | sucrose | 6.368 | 3.45×10^-3^ | ↑** |
| 22 | α-ketoglutarate | 6.596 | 8.36×10^-3^ | ↑** |
| 23 | trans-4-hydroxy-L-proline | 7.255 | 2.38×10^-11^ | ↑*** |
| 24 | 1,5-anhydroglucitol | 7.5 | 2.47×10^-8^ | ↑*** |
| 25 | glucose-1-phosphate | 8.397 | 6.06×10^-10^ | ↑*** |
| 26 | citric acid | 8.579 | 3.39×10^-7^ | ↑*** |
| 27 | aspartate | 10.86 | 1.39×10^-15^ | ↑*** |
| 28 | inosine 5'-monophosphate | 15.74 | 3.13×10^-12^ | ↑*** |
| 29 | aspartic acid | 20.62 | 1.09×10^-11^ | ↑*** |
| 30 | sarcosine | 22.06 | 1.82×10^-12^ | ↑*** |
| 31 | 1-monopalmitin | 39.48 | 6.24×10^-3^ | ↑** |

Compared with the control group, ^*^ P<0.05, ^* *^ P<0.01, ^* * *^ P<0.001, n=9; ↓: Downregulation; ↑: Up regulation; FC: Fold Change, the multiple of changes, is the ratio of high to high digits of the corresponding two groups of metabolite peaks; P-value is Kruskal Wallis non parametric test result.

**Table S2**

| **Table S2. Pathway analysis of differential metabolites** | | | | | |
| --- | --- | --- | --- | --- | --- |
| No. | Pathway Name | Total number of compounds | Number of matches | Impact | Matched metabolites |
|  |  |  |  |  | Administration group vs control group |
| 1 | Citrate cycle | 20 | 6 | 0.25782 | α-ketoglutarate；Succinate；Citrate；Pyruvate；Fumarate；Phosphoenolpyruvate |
| 2 | Alanine, aspartate and glutamate metabolism | 28 | 6 | 0.27404 | Aspartate；Citrate；Fumarate；Pyruvate；Succinate；α-ketoglutarate |
| 3 | Arginine and proline metabolism | 38 | 6 | 0.37022 | Creatine；Putrescine；Hydroxyproline；Proline；Ornithine；Pyruvate |
| 4 | Glycine,serine and threonine metabolism | 34 | 5 | 0.38193 | Glycine；Sarcosine；Threonine；Creatine；Pyruvate |
| 5 | Pyruvate metabolism | 22 | 4 | 0.20684 | Phosphoenolpyruvate；Pyruvate；Lactate；Fumarate |
| 6 | Glycolysis/Gluconeogenesis | 26 | 4 | 0.20594 | Pyruvate；Lactate；Phosphoenolpyruvate；Glucose 1-phosphate |
| 7 | Starch and sucrose metabolism | 15 | 3 | 0.36202 | Sucrose；Glucose 1-phosphate；Glucose 6-phosphate |
| 8 | Glutathione metabolism | 28 | 3 | 0.09592 | Glycine；Ornithine；Putrescine |
| 9 | Glyoxylate and dicarboxylate metabolism | 32 | 3 | 0.13757 | Citrate；Glycine；Pyruvate |
| 10 | Tyrosine metabolism | 42 | 2 | 0.16435 | Tyrosine；Fumarate；Pyruvate |

**Table S3**

| **Table S3. Glycolysis genes related to prognosis in patients with melanoma** | | | | | |
| --- | --- | --- | --- | --- | --- |
|  | Gene | HR | HR.95L | HR.95H | cox Pvalue |
| Low risk genes | ALDH3A2 | 0.74611 | 0.57811 | 0.96294 | 0.02444 |
|  | PRKAA2 | 0.78415 | 0.52550 | 1.17009 | 0.02375 |
|  | PGAM1 | 0.83156 | 0.63085 | 1.09612 | 0.03961 |
|  | MDH1 | 0.83426 | 0.55321 | 1.25810 | 0.03829 |
| High risk genes | COL5A1 | 1.16435 | 1.01408 | 1.33687 | 0.03090 |
|  | CXCR4 | 1.22650 | 1.07101 | 1.40455 | 0.00316 |
|  | CLDN9 | 1.24601 | 1.06635 | 1.45592 | 0.00563 |
|  | HIF1A | 1.27355 | 1.00241 | 1.61802 | 0.04774 |
|  | VCAN | 1.28846 | 1.11125 | 1.49392 | 0.00079 |
|  | STC1 | 1.31374 | 1.11560 | 1.54708 | 0.00107 |
|  | PLOD2 | 1.42209 | 1.15735 | 1.74740 | 0.00081 |
|  | EXT2 | 1.47698 | 1.00069 | 2.17998 | 0.04960 |
|  | LHX9 | 2.98364 | 1.01612 | 8.76087 | 0.04669 |

HR: Hazard Ratio, risk ratio, HR<1 is a low risk gene, HR>1 is a high risk gene; HR.95L and HR.95H represent the fluctuation range of the 95% confidence interval.
