# Supplementary material for: Cryptotanshinone inhibits PFK-mediated aerobic glycolysis by activating AMPK pathway leading to blockade of cutaneous melanoma
Source: Chin Med. 2024 Mar 7;19:45. doi: 10.1186/s13020-024-00913-1 (PMC10921599; doi:10.1186/s13020-024-00913-1)
Supplement: Supplementary file 7 — Additional file 7. Additional Methods. [file 13020_2024_913_MOESM7_ESM.doc]

#### Additional methods

#### Construct a data model for melanoma-related glycolytic genes to predict the prognostic biomarkers

(1) Acquisition and collation of data: download the transcriptomic data of 471 melanoma tissues and 340 normal tissues from the TCGA database (https://portal.gdc.cancer. gov/) and GTEX databases (https://xenabrowser.net/datapages/) and transform the gene names and merge the genes using Perl language. In the GSEA database (http://www.gsea-msigdb.org/gsea/msigdb/search.jsp), the gene sets related to glycolysis (HALLMARK_GLYCOLYSIS, REACTOME_GLYCOLYSIS) was retrieved with "glycolysis" and imported into GSEA 4.2.3 software. The simulation runs----permutations select 1000 times and data type is T verse N. After the run, the index file was exported to obtain the enrichment graph of glycolytic genes.

(2) Constructing a prognostic model and screening of prognostic markers: Merge the expression of glycolytic related genes with patient survival information, conduct univariate Cox regression analysis, calculate the risk ratio and 95% confidence interval of each gene to melanoma patients, and screen out glycolytic genes that are significantly correlated with the prognosis of melanoma patients under the condition of P<0.05. Using the "Survival" package in R language to conduct multi COX regression analysis on glycolytic genes related to the prognosis of melanoma, further screen prognostic related variables and obtain regression coefficients for prognostic genes, and finally construct a risk scoring equation based on glycolytic gene expression: Risk score = β1 mRNA1 EXP + β2 mRNA2 EXP + β3 mRNA3 EXP +... + βn mRNAn EXP. In the equation, β is the regression coefficient of the corresponding mRNA, mRNA EXP is the expression of the corresponding gene, and n is the relevant glycolytic gene.

(3) Survival analysis and accuracy evaluation of the prognostic model: melanoma patients were divided into high and low risk groups according to the median of patient risk values, and Kaplan-Meier survival curves of high and low risk groups were drawn using the "Survival" package in R language ^[1]^. The receiver operating characteristic curve (ROC) for 5-year overall survival rate of melanoma patients was drawn using the "Survival ROC" package in R language, and the value of the area under the curve (area under curve, AUC) was calculated to evaluate the accuracy of the model ^[2]^. The range of AUC value is generally 0.5-1.7. When it is more than 0.7, the model is considered with good accuracy.

[1] Rasmussen L, Pratt N, Hansen MR, et al. Using the "proportion of patients covered" and the Kaplan-Meier survival analysis to describe treatment persistence. Pharmacoepidemiol Drug Saf, 2018, 27(8): 867-871.

[2] Bünger R, Mallet RT. Metabolomics and Receiver Operating Characteristic Analysis: A Promising Approach for Sepsis Diagnosis. Crit Care Med, 2016, 44(9): 1784-5.
